# Quantitative trait loci associated with improved fruit yield under heat-stress conditions in fresh-market tomato

**DOI:** 10.3389/fpls.2026.1919895

**Published:** 2026-08-27

**Authors:** Bihani Thapa, Leonard M. Gaspar, Le Kang, Samuel S.F. Hutton, Jessica Chitwood-Brown

**Affiliations:** 1 Plant Breeding Graduate Program, Gulf Coast Research and Education Center, University of Florida, Gainesville, FL, United States; 2 Gulf Coast Research and Education Center, University of Florida, Gainesville, FL, United States; 3 Plant Breeding Graduate Program, Gulf Coast Research and Education Center, Horticultural Sciences Department, University of Florida, Gainesville, FL, United States

**Keywords:** fresh-market, fruit yield, heat stress, QTL analysis, tolerance, tomato

## Abstract

Rising temperatures and more frequent heat stress events pose a major challenge to global tomato production, particularly in tropical and subtropical regions such as the southern United States. High temperatures during flowering and fruit set lead to poor fruit set and reduced yield. Although several commercial cultivars and breeding lines are described as heat-tolerant, the genetic basis of yield performance under heat stress conditions in fresh-market tomato remains poorly understood. This study aimed to identify genomic regions associated with fruit yield under natural heat stress. A biparental recombinant inbred line (RIL) population developed by the UF/IFAS tomato breeding program was evaluated under natural field heat stress in the fall seasons of 2016, 2017, and 2018, with fruit yield recorded as the primary trait. Genotyping of RILs was performed with the AgriPlex commercial tomato panel. Multi-environment QTL analysis was conducted to identify loci associated with fruit yield under heat stress. A major locus on chromosome 12 was selected for validation. Backcross populations segregating for this region were evaluated in a randomized block design during the fall of 2020 at the Gulf Coast Research and Education Center (GCREC), Balm, Florida. Multi-environment QTL analysis identified several loci on chromosome 4, 5, 6, and 12 associated with fruit yield under natural heat stress conditions. Among these, a locus on chromosome 12 showed consistent effects across multiple harvests and environments and explained a relatively larger proportion of phenotypic variance. Validation using backcross populations confirmed that genotype carrying the chromosome 12 QTL produced significantly higher yield under natural heat stress than susceptible genotypes. Overall, this study identified an agronomically important region on chromosome 12 that can be targeted to improve tomato yield under heat stress. The results also highlight multiple genomic regions contributing to higher yield under heat stress. These findings provide a foundation for developing breeding strategies for developing heat-tolerant fresh-market tomato cultivars.

## Introduction

Global climate change has intensified the frequency and duration of heat stress events, posing a major threat to agricultural productivity in tropical and subtropical regions (Calvin et al., 2023). Heat stress occurs when plants experience temperatures exceeding their optimal growth and development limits, resulting in impaired physiological processes and reduced reproductive success (Wahid et al., 2007; Bita and Gerats, 2013). These effects collectively compromise plant growth and ultimately reduce crop yields, making heat stress a critical barrier to stable food production (Hatfield and Prueger, 2015; Zhu et al., 2021).

Tomato (*Solanum lycopersicum* L.) is one of the most widely cultivated vegetable crops worldwide, yet it is highly sensitive to elevated temperatures. Optimum tomato growth occurs at approximately 25-32°C during the day and 18-20°C at night, and temperatures beyond this range negatively affect both vegetative and reproductive development (Peet et al., 1998; Sato et al., 2000; Camejo et al., 2005, 2006; Zhou et al., 2017; Hoshikawa et al., 2021). The reproductive process is highly sensitive to temperatures beyond the optimal growth range (Lohani et al., 2020). High temperatures reduce pollen viability, disrupt flower development, impair photosynthetic efficiency and lead to poor fruit set, leading to significant yield losses (Firon et al., 2006; Müller and Rieu, 2016; Paupière et al., 2017; Graci and Barone, 2023; Kim et al., 2025). These challenges are particularly concerning in production systems such as Florida’s fresh-market tomato industry, where daily maximum temperatures frequently exceed 32^0^C and daily minimum temperatures remain above 20^0^C from August to mid-October in open-field environments, exposing plants to heat stress during the key developmental stage (Ayankojo & Morgan, 2020; FAWN, 2026).

Marketable yield reduction is the most consequential outcome of heat stress, and developing cultivars that maintain high productivity under elevated temperatures remains a major breeding priority. The UF/IFAS tomato breeding program has released several fresh-market cultivars with improved fruit set and production under high-temperature conditions (Scott et al., 1989, 1995a, 1995b, 1997, 2006; Hutton et al., 2020). Despite progress in developing heat-tolerant cultivars, breeding for heat tolerance in tomato remains challenging due to the complex quantitative nature of the trait, influenced by multiple physiological and developmental processes, and a limited understanding of the genetic basis underlying yield stability under heat stress (Ayenan et al., 2019). The reproductive stage is more sensitive to high temperatures and directly impacts fruit set and production; many attempts have been made to uncover the genetic basis of reproduction-related traits under heat stress. Early work identified quantitative trait loci (QTLs) for fruit set under heat stress (Grilli et al., 2007) and mapped loci on chromosomes 2, 3, 4, and 5 associated with floral and fruit traits under high temperature (Lin et al., 2010). Xu et al. (2017a) further reported QTLs on chromosomes 1, 2, 3, 7, 8, and 11 for pollen viability, stigma and style traits, and other reproductive responses to heat. Subsequent studies expanded this framework: Ruggieri et al. (2019) evaluated tomato landraces under high temperature and identified SNPs co-localizing with previously reported gene for floral development; Gonzalo et al. (2020) and Gonzalo et al. (2022) identified temperature-specific QTLs for pollen viability, fruit number, fruit set, and stigma exertion across multiple heat regimes; Bineau et al. (2021) mapped heat-responsive and plasticity QTLs for yield-related traits in MAGIC and core collection panels; Wen et al. (2019) identified QTLs for heat tolerance on multiple chromosomes using combined QTL mapping, QTL-seq and RNA-seq approaches. Recent genomic work in a heat-tolerant line identified candidate genes, some of which mapped to QTLs associated with flowering and included heat shock proteins (HSPs), heat shock factors (HSFs), and others involved in flowering and pollen activity (Graci et al., 2023, 2024). Together, these studies on reproduction-related traits have provided a critical foundation for understanding heat tolerance in tomato. However, most of these studies have relied on individual reproductive components and were conducted under controlled greenhouse conditions; notably pollen viability alone has been shown to be an insufficient predictor of heat tolerance and yield performance in some cultivated germplasm (Miller et al., 2021). This creates a gap in linking these processes to fruit production and yield under natural field heat-stress, where yield serves as a direct and integrative measure of reproductive success (Xu et al., 2017b; Diouf et al., 2020). As a result, the relevance of these loci to open-field production systems, where plants experience long-term, naturally occurring heat stress combined with additional biotic and abiotic factors, remains unclear. In addition, many existing genetic studies have been conducted on small-fruited, processing, or wild germplasm rather than large-fruited fresh-market tomato germplasm (Driedonks et al., 2018; Bashary et al., 2024), and alleles identified in different genetic backgrounds may not segregate or express the same effects in fresh-market cultivars, limiting their direct utility for breeding programs for large-fruited, marketable yield. This gap limits the development of molecular tools needed for breeding heat-tolerant fresh-market tomato cultivars and highlights the need for field-validated genetic studies in fresh-market germplasm adapted to high-temperature production environments.

To address this gap, we conducted QTL mapping in a recombinant inbred line (RIL) population derived from a cross between a heat-tolerant and a heat-susceptible fresh-market tomato parent, evaluated across three fall seasons under naturally occurring heat stress in open-field conditions in Florida. The objective of this study was to identify genomic regions associated with fruit yield under heat stress and validate their contribution to yield performance in backcross populations. This approach was designed to provide field-validated, breeding-relevant genetic targets to improve heat tolerance in fresh-market tomato germplasm adapted to warm production environments.

## Materials and methods

### Plant materials

A RIL population was developed from a cross between the heat-tolerant tomato breeding line Fla. 8044 and the heat-susceptible line Fla. 8059. The two parental lines were selected based on their contrasting responses to high temperatures during fruit set. The RIL population consisted of 154 lines advanced to the F6 generation using the single-seed descent method. Seeds of parental lines and RILs were maintained and increased under field conditions at the UF/IFAS Tomato Breeding Program, Gulf Coast Research and Education Center (GCREC), Wimauma.

### Field evaluation of the RIL population

#### Experimental site, experimental design, and growing conditions

**Figure 1:**
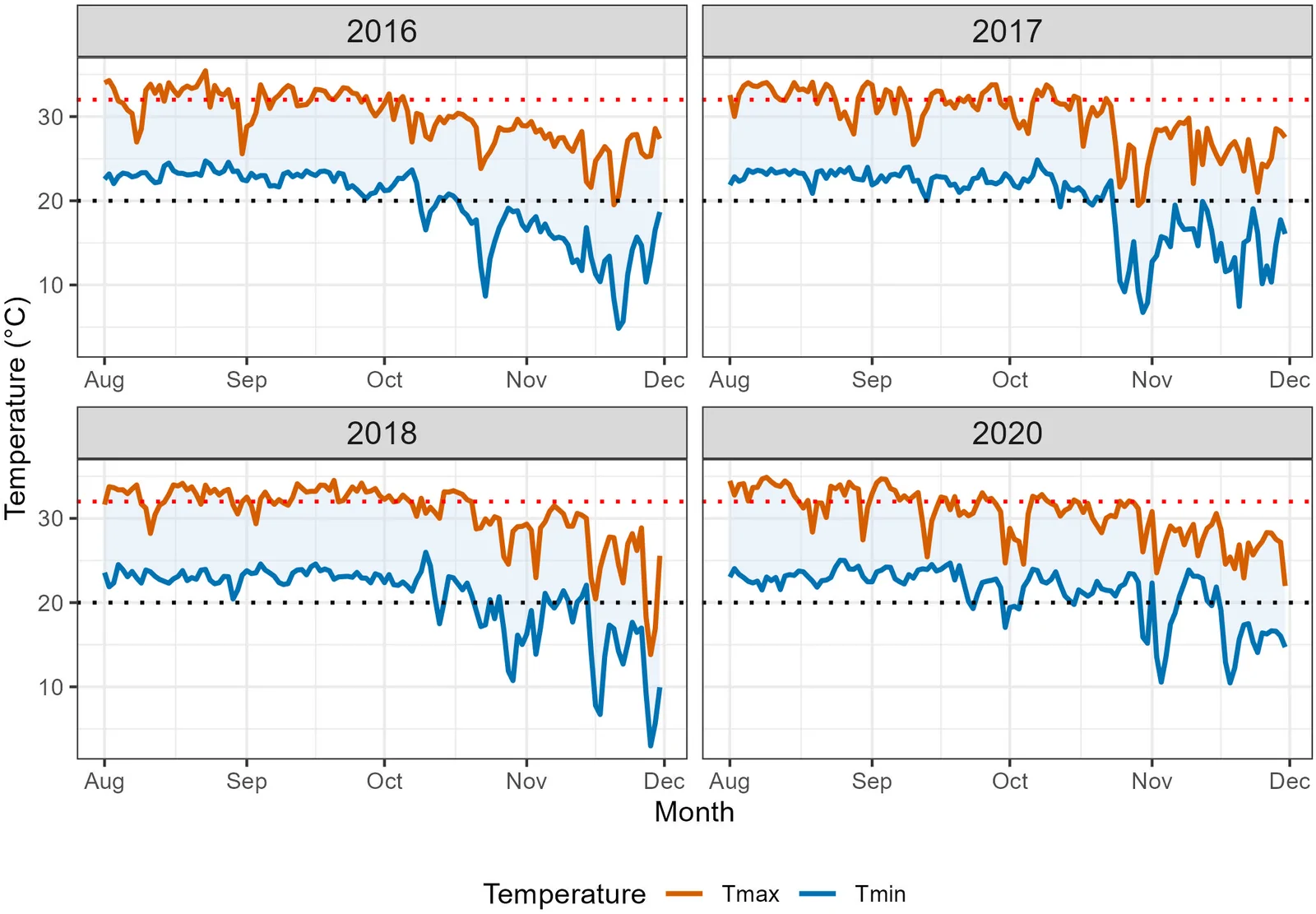
Daily maximum (Tmax) and minimum (Tmin) temperatures during fall season (August-November) for 2016, 2017, 2018 and 2020 in Balm, Florida. The shaded region represents the range between daily minimum and minimum temperatures. Horizontal dashed lines indicate temperature threshold of 20 ^0^C (Black) and 32 ^0^C (red), highlighting potential heat stress conditions. Temperature data were obtained from the Florida Automated Weather Network (FAWN).

Field trials were conducted at the GCREC in Wimauma, Florida, during the fall growing seasons of 2016, 2017, and 2018. The site is characterized by sandy soil and a subtropical climate with naturally occurring high temperatures during the early growth and developmental stages. Daily maximum and minimum temperatures were obtained from the Florida Automated Weather Network (FAWN) database for the entire growing season to characterize heat stress conditions. These temperature data are summarized and presented in **Figure 1**. Temperature thresholds of 32^0^C and 20^0^C were used as reference values for daily maximum and minimum temperatures, respectively, based on critical limits above which tomato reproductive processes including pollen viability, flower retention and fruit set are adversely affected (Peet et al., 1998; Sato et al., 2000). The RIL population, along with the two parental lines and additional check entries, was evaluated using a randomized complete block design (RCBD) with two replications. A total of 162 entries were included in the field evaluation, comprising 154 RILs, the two parental lines (Fla. 8044 and Fla. 8059), and 6 additional check entries. A plot consisting of nine plants was used as an experimental unit. Seedlings were grown in 128-cell propagation trays filled with peat-lite mix and transplanted at five weeks of age at 45 cm spacing into plastic-mulched field beds (20 cm high and 80 cm wide). Standard agronomic practices recommended for Southwest Florida, including irrigation, fertilization, staking, and disease pest management, were followed for the duration of the growing season.

#### Phenotyping

Fruit yield was the primary trait considered for the field evaluation of the RIL population. Five plants per plot were harvested, and five harvests were made at weekly intervals during the production season. At each harvest, the number and weight of fruit were recorded. The sum of all harvests was used to calculate the total fruit yield. To capture temporal variation in fruit production, yield was portioned into stage-specific components based on yield accumulated during specific harvest windows expressed in days after transplanting (DAT): early-season fruit yield (≤90 DAT), mid-season fruit yield (91–100 DAT), and late-season fruit yield (>100 DAT).

#### Genotyping

Young leaf tissue was collected from each RIL and the two parental lines and sent to AgriPlex genomics for genotyping with Commercial tomato panel, which contains 1039 genome-wide SNP markers. Of the 154 RILs, 12 were excluded from further analysis due to genotyping failure, resulting in a final mapping population of 142 RILs used for linkage map construction and QTL analysis.

#### SNP filtering and binning

Raw SNP genotype data were first processed using the SNP function of QTL IciMapping, and marker filtering was performed based on parental and progeny information. SNP markers were removed if they met any of the following criteria: (i) missing genotype in all parents, (ii) absence of polymorphism between parents, (iii) lack of polymorphism in the RIL population. For markers with missing data in only one parent, the missing parental genotype was imputed based on the segregation pattern observed in the progeny. Following marker filtering, the remaining markers were subjected to redundancy removal using the BIN function in QTL IciMapping. Redundant markers showing identical segregation patterns were grouped into recombination bins, with physical anchor information used to guide binning. Missing values were considered during the binning process to ensure accurate identification of marker redundancy. One representative marker per bin was retained for linkage map construction, while co-segregating markers were assigned the same genetic position. The filtered and binned marker dataset was subsequently used to construct a genetic linkage map.

#### Linkage map construction

A genetic linkage map was constructed in QTL IciMapping (Li et al., 2007; Meng et al., 2015) using the MAP module, which implements the standard method for linkage map construction in biparental populations. It involves three general steps: Grouping of markers, ordering them within a chromosome, and Rippling. Markers were grouped into chromosomes based on physical anchor information. Marker orders within each chromosome were fixed according to their physical positions using the Anchor order option to preserve collinearity between the genetic and physical maps. Genetic distances were calculated using the Kosambi mapping function. Rippling was not performed as marker order was predefined by anchoring information, and further re-ordering could disrupt physical collinearity. The resulting linkage map showed biologically realistic chromosome lengths and recombination patterns and was used for downstream QTL analysis.

### QTL analysis

#### Phenotype data analysis

All statistical analyses were performed using R. Fruit yield was analyzed using a linear mixed model to obtain best linear unbiased estimates (BLUEs) for each genotype. The model was specified as:


$$Y_{ijk}=\mu +Year_{K}+\;Genotype_{i}+{\left(Genotype\times Year\right)}_{ik}+Block_{j(k)}+\epsilon_{ijk}$$

Where *Y_ijk_* is the observed yield of genotype *i* in block *j* during year *k*; m is the overall mean; Year is treated as a fixed effect to account for environmental differences among years; Genotype is treated as a fixed effect to estimate BLUEs; Block nested within year is treated as a random effect, and *e_ijk_* represents the residual error. BLUEs were preferred over best linear unbiased predictions (BLUPs) as they estimate observed genotypic performance without shrinkage toward the population mean, making them more appropriate for QTL mapping than BLUPs, which are optimal for predicting genetic potential when genotypes are treated as a random sample from a breeding population. The model was fitted using restricted maximum likelihood (REML). After model fitting, standard diagnostic checks were performed to verify assumptions of normality, homoscedasticity, and independence of residuals. Year-specific phenotypic BLUEs were extracted. Phenotypic data from all 162 field entries were included in statistical analysis; however, phenotypic BLUEs of only the 142 successfully genotyped RILs were used for QTL mapping.

### QTL mapping approach

QTL mapping was conducted using QTL IciMapping (Meng et al., 2015) in a RIL population. Year-specific phenotypic BLUEs for each trait were used as phenotypic inputs for QTL mapping to evaluate the consistency and stability of QTL across years. Inclusive Composite Interval Mapping for Additive Effects (ICIM-ADD) was used to identify QTL positions by controlling background genetic noise and improving mapping precision. Genome-wide LOD thresholds were determined using 1000 permutation tests at a = 0.05. Multi-environment QTL analysis (MET) was conducted for each fruit production trait by treating year as an environment. For each QTL detected, additive effects, LOD score, and percentage of phenotypic variance explained (PVE) were calculated across years as well as QTL-year interactions.

### QTL validation study

#### Development of the BC1F2 and BC2F2 populations

To validate the major QTL identified in the RIL population, a BC1F2 population was developed. A selected RIL (HTRIL-076) carrying the favorable allele at the target QTL was crossed with the recurrent parent Fla. 8059 to generate BC1F1 plants, which were selfed to produce BC1F2 progeny. Furthermore, BC1F2 progeny with the chromosome 12 QTL were again crossed with the recurrent parent Fla. 8059 to generate BC2F1 plants, which were selfed to produce BC2F2 progeny. A total of 768 individuals were genotyped using two flanking markers of the target QTL on chromosome 12. BC1F2 and BC2F2 plants were classified into three genotypic groups based on marker genotype: tolerant homozygous (QTL/QTL), heterozygous (QTL/-), and susceptible homozygous (-/-).

#### Field evaluation

The recurrent and donor parents of the RIL population, HTRIL-076, and three backcross populations derived from HTRIL-076 (BC1F1, BC1F2, and BC2F2) were evaluated under field conditions at GCREC during fall 2020. The experiment was carried out in an RCBD with eight replications. Five harvests were made at weekly intervals during the production season. Harvesting, fruit yield recording, and yield categorizations were performed as described in the phenotyping section above. Fruit yield in kilograms (Kg) from five plants per plot were used for data analysis.

#### Statistical analysis for QTL validation

To estimate genotype performance and access genotypic effects, fruit yield data were analyzed using a linear mixed model that accounted for block structure. The model was specified as:


$$Y_{ij}=\mu +Genotype_{i}+Block_{j}+\epsilon_{ij}$$

Where *Y_ij_* is the observed yield of genotype *i* in block *j*; µ is the overall mean; Genotype is treated as fixed effect to estimate genotypic effects; Block is treated as a random effect and *e_ijk_* represents the residual error. The model was fitted using restricted maximum likelihood (REML). Genotype BLUEs were extracted from the fitted model. Tukey’s HSD test was conducted to assess significant differences among the mean values of the genotypes.

## Results

### Phenotypic variation for fruit production traits

**Figure 2:**
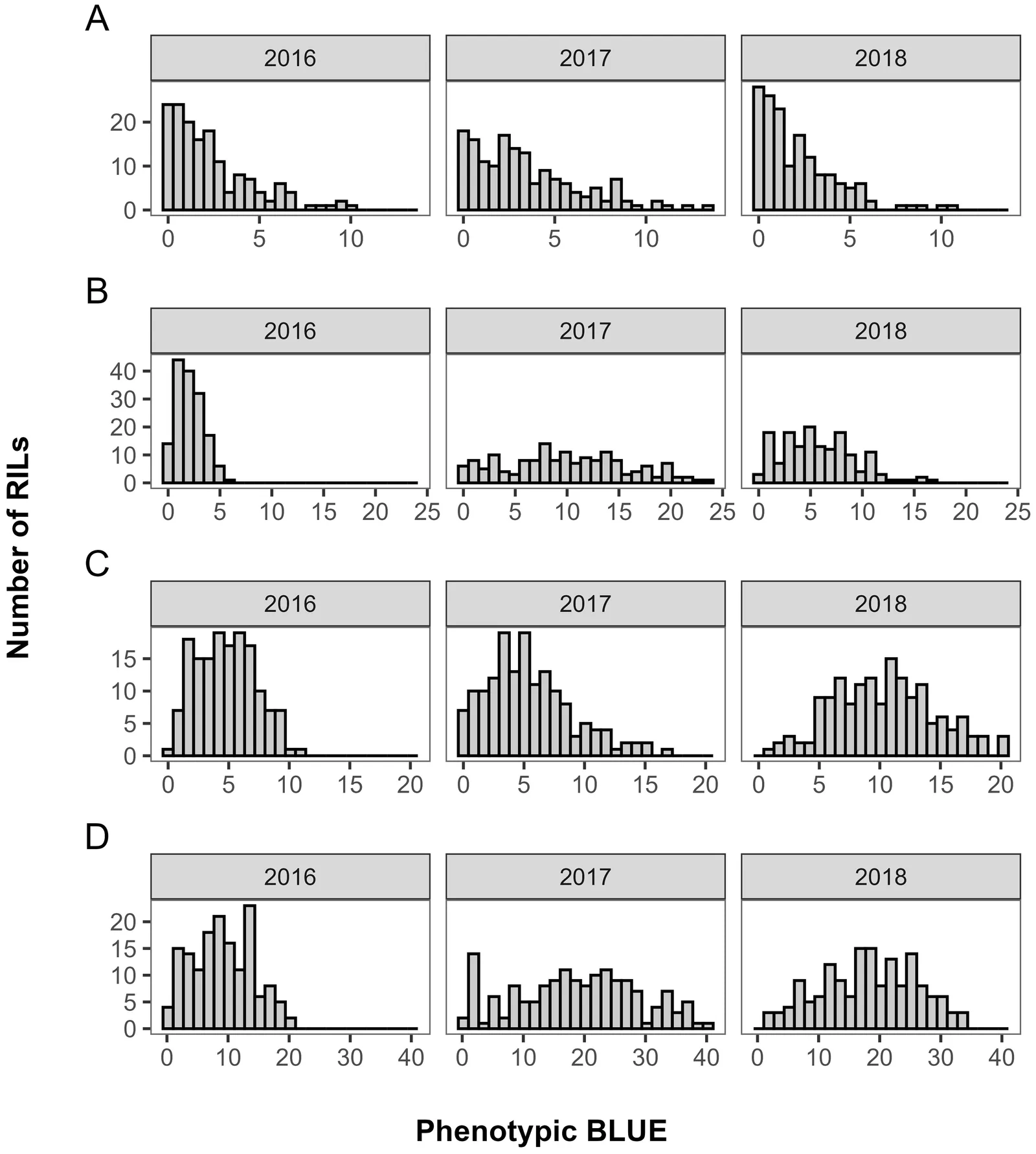
Distribution of phenotypic best linear unbiased estimates (BLUEs) for fruit yield (kg/5plants) across fall seasons of 2016, 2017, and 2018. Each panel displays a histogram of BLUEs for yield accumulated during specific harvest windows expressed in days after transplanting (DAT) for RILs **(A)** early-season fruit yield (≤90 DAT), **(B)** mid-season fruit yield (91–100 DAT), **(C)** late-season fruit yield (>100 DAT), and **(D)** Total fruit yield (cumulative fruit yield). The x-axis shows phenotypic BLUE values, and the y-axis indicates the number of RILs.

Large phenotypic variation was observed among RILs for fruit yield across different harvest windows throughout the growing season, indicating a strong genetic basis for fruit production (**Figure 2**). Fruit yield was evaluated as early-season, mid-season, late-season, and total fruit yield to capture both temporal and cumulative patterns of fruit production under heat stress. Mixed-model analysis showed that genotype effects were highly significant for all yield traits, indicating the presence of significant genetic variation that controls fruit production throughout the growing season. Year effects were also significant, highlighting the influence of environmental conditions on yield expression. In addition, significant line-by-year interactions were observed for all fruit yield categories, suggesting differential performance of genotypes across years and emphasizing the importance of multi-year evaluation. (**Supplementary Table 1**). Model fit was evaluated using residual diagnostics, which showed model assumptions were reasonably met (**Supplementary Figures S1**–**S4**). Residual histograms and density plots were centered around zero with approximate symmetry, while Q-Q plots showed minor deviations at the tails, particularly for early and mid-season yield. These deviations in yield data are expected and do not indicate a severe violation of normality.

Adjusted phenotypic values (BLUEs) were calculated for fruit production at each harvest window using a linear mixed model. The resulting BLUE distributions differed among yield categories and showed distinct patterns of variation. Early fruit yield showed a skewed distribution, with most lines producing low yield and a smaller number showing higher early productivity. In contrast, mid- and late fruit yields exhibited broader, more balanced distributions, reflecting greater variation among the RILs during later stages of fruit development and production. Total fruit yield had the widest range of values and followed an approximately symmetrical distribution (**Figure 2**). Overall, the observed phenotypic diversity, distinct distribution pattern at each harvest window, and adjusted trait values (phenotypic BLUEs) provide a suitable foundation for QTL mapping.

### Linkage map construction

A genetic linkage map was constructed using SNP markers that were polymorphic in the parents and informative in the RIL population. Parental lines and RILs were initially genotyped using the AgriPlex commercial tomato panel, which consists of 1039 SNPs. After marker quality assessment and redundancy reduction, 191 SNP markers were retained for genetic map construction. Marker position and detailed map information are provided in (**Supplementary Figure 5**). The final linkage maps spanned 1045 cM, with individual chromosome lengths ranging from ~21 to ~151 cM (**Table 1**). The number of markers per chromosome ranged from 6 to 36, with chromosomes 1, 5, and 8 showing the highest marker densities (**Table 1**). Despite variation in marker density and recombination patterns, all chromosomes were represented by ordered markers spanning their genetic lengths. Markers within each chromosome were ordered based on their physical positions in the reference genome, resulting in coherent linkage groups across all 12 chromosomes. Several regions showed marker clustering, reflecting limited recombination, while other regions were more sparsely populated. Together, this linkage map provided genome-wide coverage and supported the use of linkage maps for QTL detection of fruit yield across harvest windows and years.

**Table 1:** Summary of the genetic linkage map constructed from the RIL population used in this study.

Chromosome	No. of marker	Genetic length (cM)
1	30	150.62
2	12	99.44
3	17	52.59
4	12	110.72
5	25	126.53
6	7	66.41
7	14	113.73
8	36	82.9
9	12	105.88
10	8	77.49
11	6	20.97
12	12	37.89
**Total**	**191**	**1045.17**

Bold values represent the total number of markers and the total genetic length across all chromosomes.

### QTL analysis of fruit yield traits

#### Multi-environment QTL analysis

QTL analysis across years identified several genomic regions associated with early, mid, late, and total fruit yield (**Table 2**). Using a multi-environment analysis approach enabled the identification of loci with consistent genetic effects and those whose effects varied across years. QTLs associated with fruit yield under heat stress conditions were detected on chromosomes 4, 5, 6 and 12. Individual QTLs explained between 3.1 and 14.6% of the phenotypic variance, reflecting a complex genetic architecture underlying fruit yield under heat stress. For early fruit yield, two QTLs were detected on chromosome 6 (~66cM) and chromosome 12 (~23cM) (**Table 2**). The chromosome 12 QTL explained a relatively greater proportion of phenotypic variance (~14%) and was primarily controlled by additive effects, indicating stable genetic contribution to early fruit production under high temperatures. The chromosome 6 QTL showed a smaller and less consistent effect across years, as indicated by a modest A×E interaction, suggesting that the contribution of the favorable allele at this locus depends on the heat-stress conditions experienced during early plant development. Four QTLs were identified on chromosomes 4, 5, 6 and 12 for mid-season fruit yield, explaining moderate proportions of phenotypic variance (~5-10%) (**Table 2**). Several of these loci showed detectable A×E interactions. For late-season fruit yield, four QTLs were detected on chromosomes 5, 6 and 12 (**Table 2**). The QTL on chromosome 6 (~66 cM) showed a comparatively strong effect, explaining nearly 14% of the phenotypic variance, with a notable A×E interaction. For total fruit yield, four QTLs were identified on chromosomes 5, 6 and 12, among which the QTL on chromosome 12 explained the highest phenotypic variance (14.6%). QTL validation.

**Table 2:** QTLs detected for the fruit yield during different harvest windows in field experiments conducted in fall seasons of 2016, 2017 and 2018 using multi-environment QTL analysis (MET) with ICIM additive approach in QTL ICIMapping.

Trait	Chr	Genetic position (cM)	Left marker	Right marker	LOD	LOD (A)	LOD (AbyE)	PVE	PVE (A)	PVE (AbyE)	Add
Early-season fruit yield	6	66	UFTLP4-6333	UFTLP1_2059	7.3	6.8	0.4	8.7	7.3	1.4	0.6
	12	23	solcap_snp_sl_7042	solcap_snp_sl_7045	12.7	12.2	0.4	14.4	13.4	0.9	0.8
Mid-season fruit yield	4	33	UFTLP4-1277	solcap_snp_sl_11543	4.9	3.7	1.2	6.6	4.8	1.8	-0.8
	5	123	UFTLP1_1961	UFTLP1_1975	4.9	3.2	1.7	5.2	4.4	0.8	0.7
	6	65	UFTLP4-6333	UFTLP1_2059	4.6	2.6	2.1	5.2	3.5	1.7	0.7
	12	29	solcap_snp_sl_19632	solcap_snp_sl_31973	9.3	5.6	3.7	9.8	8.0	1.9	1.0
Late-season fruit yield	5	115	solcap_snp_sl_16204	UFTLP1_1961	4.1	1.1	3.0	3.8	1.7	2.1	0.3
	6	66	UFTLP4-6333	UFTLP1_2059	7.9	4.6	3.3	13.8	7.1	6.6	0.7
	12	23	solcap_snp_sl_7042	solcap_snp_sl_7045	4.7	4.4	0.3	9.4	6.7	2.7	0.7
	12	37	solcap_snp_sl_31973	UFTLP1_3611	5.0	3.5	1.6	5.4	5.3	0.1	0.6
Total fruit yield	5	124	UFTLP1_1961	UFTLP1_1975	4.3	3.4	0.9	4.7	3.6	1.2	1.3
	6	65	UFTLP4-6333	UFTLP1_2059	8.8	7.8	1.0	9.9	9.0	0.9	2.1
	12	23	solcap_snp_sl_7042	solcap_snp_sl_7045	11.5	10.7	0.8	14.6	12.2	2.4	2.5
	12	34	solcap_snp_sl_31973	UFTLP1_3611	6.7	2.1	4.6	3.1	2.3	0.8	1.1

Genetic position of QTLs on a chromosome and flanking markers information is presented. Other statistical estimates for the detected QTLs include: LOD for maximum LOD score, LOD (A) for additive effects, LOD (AbyE) for additive effects and year interaction, and similar estimates with percent variance explained (PVE).

**Figure 3:**
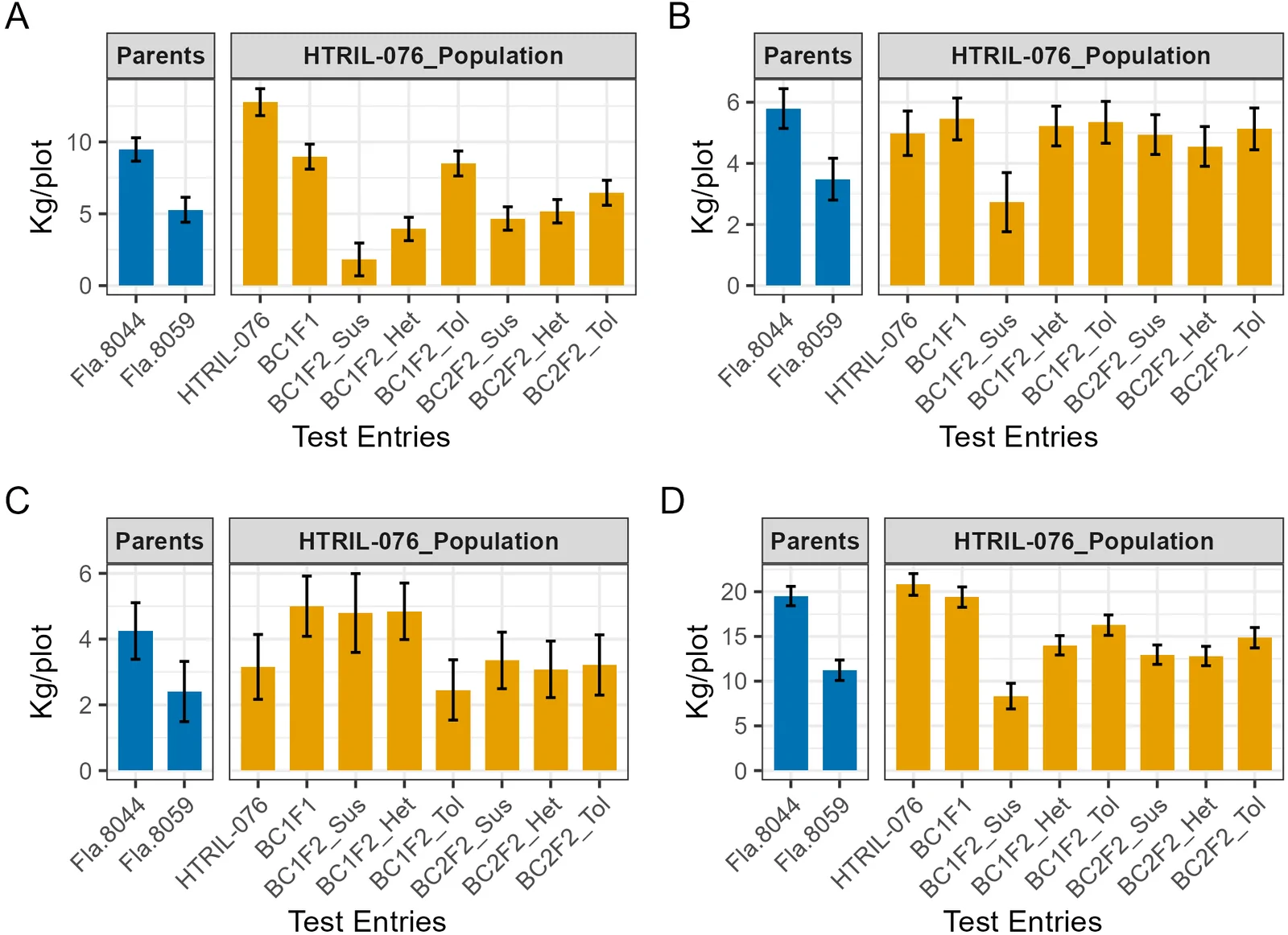
Estimated marginal means of fruit yield for parental lines and entries from the BC1F2 and BC2F2 validation populations carrying tolerant, heterozygous or susceptible alleles at the chromosome 12 region. Panels show **(A)** early-season fruit yield, **(B)** mid-season fruit yield, **(C)** late-season fruit yield and **(D)** total fruit yield. Yield Kg/plot represents the fruit yield from five plants. Error bars represent ± standard error of the estimated marginal means.

Mixed-model analysis showed that genotype had a clear effect on early-season fruit yield and total fruit yield, while effects were not significant for mid-season and late-season fruit yield (**Supplementary Table 2**). Estimated marginal means showed clear separation among allelic classes in the BC1F2 generation (**Figure 3**). For early-season fruit yield, BC1F2 entries carrying the tolerant allele at the chromosome 12 QTL region produced higher yields than susceptible entries. Heterozygous entries generally showed intermediate values. A similar pattern was observed for total fruit yield, consistent with the strong overall genotype effect detected by the mixed model analysis (**Supplementary Table 2**). Differences among the BC1F2 allelic classes were smaller for mid-season fruit yield and less consistent for late-season fruit yield (**Figures 3b, c**). In the BC2F2 generation, tolerant entries showed higher mean fruit yield than susceptible entries across yield categories (**Figure 3**). However, these differences were smaller than those observed in BC1F2 and did not reach statistical significance (**Supplementary Table 3**). Overall, Yield differences were most distinct in the BC1F2 generation and were strongest during early fruit production and total yield (**Figure 3**).

## Discussion

Heat stress during fall production is a major constraint on fresh-market tomato production, as exposure to high temperatures during flowering and early fruit development often leads to poor fruit set and reduced yield. These losses arise from disruptions to key physiological and reproductive processes, including pollen viability, flower retention, and fertilization success (Dane et al., 1991; Peet et al., 1998; Sato et al., 2000; Firon et al., 2006; Nankishore and Farrell, 2016; Ayenan et al., 2021; Lee et al., 2022). Because fruit yield integrates the cumulative effects of these disruptions across the growing season, it represents a more direct measure of performance under heat stress than individual reproductive proxies (Miller et al., 2021). In this context, this study aimed to identify the genetic basis of increased fruit yield under heat-stress conditions. We conducted QTL mapping using a RIL population evaluated exclusively during fall seasons when transplanting in early August ensured that vegetative growth and flowering coincided with naturally occurring high temperatures and validated selected loci in backcross populations. Multiple QTLs associated with early-, mid-, and late-season fruit yield, as well as total fruit yield, were identified, with a locus on chromosome 12 showing consistent effects across years and QTL validation populations. While yield is known to be a polygenic trait (Cappetta et al., 2021), this study provides field-based evidence of how specific genomic regions contribute to increased yield under heat stress and how their effects vary with genetic background, offering practical insights into selection strategies for tomato breeding in warm production environments.

The most significant QTL for early-season and total fruit yield in this study was located on chromosome 12 and explained 14.4% of the phenotypic variation under natural heat stress. This region, mapped near 23cM between markers solcap_snp_sl_7042 and solcap_snp_sl_7045 (~63.3 Mb), also showed a strong effect on total fruit yield (14.6% PVE) and was consistently detected across years, indicating a stable effect for these yield components. Additional QTL for mid-season, late-season, and total yield were detected at 29–37 cM (**Table 2**), all mapping to the same physical interval (~63–65 Mb), indicating a QTL cluster or a single pleiotropic locus influencing yield throughout the season. Previous studies have also reported QTLs on chromosome 12 associated with reproductive traits under heat stress, such as pollen viability, fruit set, and yield; however, these loci were positioned in different regions of the chromosome (Xu et al., 2017a; Wen et al., 2019; Gonzalo et al., 2020; Bineau et al., 2021; Elazazi et al., 2024). The chromosome 12 region identified in this study falls within the distal portion of the broad fruit-weight QTL FW12.2, which spans more than 57 Mb on the tomato reference genome SL4.0 (Bhandari et al., 2023). Within this interval, Bhandari et al. (2023) reported two small fruit yield QTL (mID525 and mID558), with mID558 overlapping with our early-season and total fruit yield QTL. However, this region does not overlap any of the cloned domestication genes that control fruit weight and shape, such as FW2.2, FW3.2, LC, FAS, SUN, and OVATE, as summarized by van der Knaap et al. (2014), nor does it correspond to the fruit weight QTL identified during domestication transitions (Pereira et al., 2021). This suggests that the locus represents an additional yield-related region segregating in modern fresh-market germplasm. The consistent effects across early-, mid-, and late-season, as well as total yield, indicate that the favorable allele is associated with higher yield performance under the high temperature stress conditions evaluated in this study. Collectively, these results identified an agronomically important region on chromosome 12 that contributes to improved yield under heat-stress conditions in fresh-market germplasm.

In addition to the major chromosome 12 locus, a few additional QTLs were detected on chromosomes 4, 5 and 6 for fruit yield across different harvest windows (**Table 2**). These loci explained a moderate proportion of phenotypic variation (3-10% PVE) and represent additional genomic regions that may contribute to yield under high-temperature stress. Notably, Bineau et al. (2021) identified QTLs on chromosomes 4, 5, 6, and 12 for yield and reproductive traits in MAGIC and core collection populations evaluated under heat stress conditions. Similarly, Elazazi et al. (2024) identified QTLs on chromosomes 5, 6, and 12 associated with improved fruit set and fruit weight under high-temperature conditions. Although the specific intervals we identified do not overlap with QTLs reported by previous studies on tomato yield and reproductive traits under heat stress (Xu et al., 2017a; Gonzalo et al., 2020, 2022; Bineau et al., 2021; Elazazi et al., 2024), positional differences are expected given the divergent germplasm backgrounds and traits used across studies (Diouf et al., 2020). This is further expected because previous studies relied on wild or highly diverse germplasm and focused on reproductive or fruit set traits, which serve as indirect proxies for heat tolerance and yield potential under stress conditions (Xu et al., 2017b). In contrast, our study evaluated fruit production in a RIL population derived from modern fresh-market breeding lines, demonstrating that this yield-associated region segregates within commercially relevant germplasm and contributes to variation in yield under heat-stress conditions.

The validation study supports the contribution of the chromosome 12 region to fruit yield under high-temperature field conditions. Mixed-model analysis showed genotype effects on early-season and total fruit yield in the BC1F2 generation, with entries carrying the tolerant allele producing significantly higher yields than heterozygous or susceptible entries. The higher yield observed in lines carrying the tolerant allele indicates improved reproductive success and fruit set under heat stress, traits widely associated with heat tolerance and yield stability in tomato (Grilli et al., 2007; Lee et al., 2022). In the BC2F2 generation, a weaker effect of the tolerant allele was observed for fruit yield and mid and late season yield differences were not statistically significant in either generation. This likely reflects differences in the genetic background between the validation populations. BC2F2 entries contain a larger proportion of the susceptible parent genome, and reduced support from complementary loci can reduce the phenotypic effect of a target region in later backcross generations (Gur and Zamir, 2004; Lecomte et al., 2004; Semel et al., 2006). It is also important to note that classification of BC1F2 and BC2F2 entries was based solely on marker genotype at the chromosome 12 region and alleles at other identified QTLs were not controlled during selection. As a result, individuals within the same chromosome 12 allelic class may differ at additional loci influencing fruit weight and yield. This genetic heterogeneity may have increased within-class variation, resulting in larger standard errors and making it difficult to detect significant effects between classes. This may also indicate that the magnitude of the effect of the QTL on chromosome 12 is influenced by the broader genetic background. Despite these complexities, the validation results provide supportive evidence for the association of the chromosome 12 locus with early and total fruit yield under heat stress conditions.

The chromosome 12 QTL interval (~63.2- 65.0 Mb; SL4.0) is a gene-dense region containing more than 150 annotated genes based on the SL4.0 reference genome assembly and ITAG4.0 annotation, the majority with predicted functions and 12 annotated as unknown proteins. A preliminary survey identified several candidates with putative roles in heat stress response and reproductive development. These include Solyc12g088890.3, encoding a DnaJ-like protein, a family of molecular chaperones members of which have been associated with thermotolerance in tomato (Wang et al., 2019). The interval also contains Solyc12g089350.3, encoding a GDSL esterase/lipase, a gene family implicated in heat stress response in tomato (Graci et al., 2024). Additionally, Solyc12g089040.3 a mature anther-specific protein (LAT61) expressed during pollen maturation, relevant given the sensitivity of anther development and pollen viability to heat stress and their direct influence on fruit set (Peet et al., 1998; Sato et al., 2000). Other genes encoding sugar facilitator protein 6 (Solyc12g089180.2) and catalase (Solyc12g094620.3) can be additional candidates given the known importance of carbohydrate metabolism and antioxidant enzyme activity under heat stress (Wahid et al., 2007). Given the large physical size of the chromosome 12 QTL interval and the number of annotated genes it contains, fine mapping will be necessary to narrow the confidence interval and prioritize candidates for functional validation. This work is currently underway.

QTL mapping was performed using a single biparental population, which limits the allelic diversity represented (Ayenan et al., 2019). Although the initial genotyping platform contained 1039 SNP markers, only 191 markers were retained for linkage map construction. The resulting map provided sufficient genome coverage for QTL detection; however, the relatively modest marker density may limit precise localization of causal loci, and some QTLs may remain undetected in sparsely covered genomic regions. This limitation is also relevant for chromosome 12, where multiple QTLs associated with different harvest windows were detected within a relatively narrow genomic interval. At the current marker density, it remains unclear whether these signals represent multiple linked loci with independent effects on yield across the season or a single pleotropic region influencing yield throughout the growing season. Fine-mapping with higher marker density will be necessary to resolve this ambiguity.

All evaluations were conducted under fall field conditions in Florida; therefore, the stability of the identified QTLs across different environments, growing seasons, and heat stress intensities remains to be determined (Tiwari et al., 2013; Diouf et al., 2020). Furthermore, the absence of a non-stress control environment limits the ability to determine whether the identified loci are specifically associated with heat stress responsiveness or reflect more general yield potential. In addition, the focus on fruit production did not address other physiological or molecular processes associated with heat tolerance that may influence reproductive performance. Although validation confirmed the effect of the chromosome 12 locus, segregation at other genomic regions likely contributed to increased variation and reduced statistical power in evaluating the different backcross populations. Nevertheless, the results identify genomic regions relevant to improving tomato yield performance under heat stress and provide a foundation for further validation across diverse genetic backgrounds and environments.

Overall, this study advances understanding of the genetic basis of yield performance under heat stress in fresh-market tomato and pinpoints genomic regions that can be integrated into breeding efforts to improve tomato productivity under high-temperature production conditions. Given the increasing frequency of high-temperature events in major tomato-growing regions worldwide, deploying these QTLs through marker-assisted or genomic selection breeding approaches could improve production stability (Cappetta et al., 2021). Future work will focus on fine-mapping the promising loci, particularly the chromosome 12 region, to identify candidate genes and their functions. Expanding QTL discovery to more diverse populations, such as MAGIC designs (Elazazi et al., 2024; Lin et al., 2025), will broaden the allelic diversity available to improve heat-resilient cultivars. Exploring other heat-response traits correlated with yield may enhance further selection accuracy and efficiency for this complex trait (Xu et al., 2017b). Incorporating the identified QTLs, particularly the chromosome 12 locus along with loci on chromosomes 4, 5, and 6 as fixed effects in genomic prediction models may improve genomic predictive ability for yield under heat stress. This would expand the utility of these findings beyond marker-assisted selection and support the development of genomic selection pipelines for fresh-market tomato yield under high-temperature production environments.

## Data Availability

The dataset generated and analyzed for this study are publicly available in the Mendeley Data repository. Thapa, Bihani; Chitwood-Brown, Jessica (2026), “Dataset for: Quantitative trait loci associated with improved fruit yield under heat-stress conditions in fresh-market tomato.”, Mendeley Data, V1, doi: 10.17632/s8b789c99b.1.
