# Supplementary material for: Quantitative trait loci associated with improved fruit yield under heat-stress conditions in fresh-market tomato
Source: Front Plant Sci. 2026 Aug 27;17:1919895. doi: 10.3389/fpls.2026.1919895 (PMC13558229; doi:10.3389/fpls.2026.1919895)
Supplement: Supplementary file 1 [file DataSheet1.docx]

Supplementary Material

# 1 Supplementary Figures and Tables

## 1.1 Supplementary Figures

Supplementary Figure S1. Residual diagnostics for the early-season fruit yield model fitted to RIL population phenotype data.

Supplementary Figure S2. Residual diagnostics for the mid-season fruit yield model fitted to RIL population phenotype data.

Supplementary Figure S3. Residual diagnostics for the late-season fruit yield model fitted to RIL population phenotype data.

Supplementary Figure S4. Residual diagnostics for the total fruit yield model fitted to RIL population phenotype data.

Supplementary Figure S5. Linkage map of the tomato recombinant inbred line (RIL) population derived from a cross between Fla. 8044 and Fla. 8059. The map spans all 12 tomato chromosomes. Genetic distances are indicated in centimorgans (cM) on the left side of each chromosome and marker names are shown on the right.

## 1.2 Supplementary Tables

**Supplementary Table S1**. Analysis of variance for early-season, mid-season, late-season and total fruit yield in the recombinant inbred line (RIL) population. Line and year were treated as fixed effects, and their interaction (Line × Year) was included in the linear mixed model fitted by REML. SS=sum of squares; MS= Mean squares; df= degree of freedom; F= F-statistics; P=probability value.

| **Source of variation** | **df** | **Early-season yield** | | | | **Mid-season yield** | | | | **Late-season yield** | | | | **Total yield** | | | |
| --- | --- | --- | --- | --- | --- | --- | --- | --- | --- | --- | --- | --- | --- | --- | --- | --- | --- |
|  |  | **SS** | **MS** | **F** | **P** | **SS** | **MS** | **F** | **P** | **SS** | **MS** | **F** | **P** | **SS** | **MS** | **F** | **P** |
| **Line** | 161 | 3839.8 | 23.85 | 8.25 | <0.001 | 9130.7 | 56.71 | 6.29 | <0.001 | 6806.9 | 42.28 | 5.37 | <0.001 | 45569 | 283.04 | 15.45 | <0.001 |
| **Year** | 2 | 93.3 | 46.63 | 16.14 | 0.025 | 1004.9 | 502.46 | 55.75 | 0.004 | 1326.7 | 663.37 | 84.31 | 0.002 | 1301 | 650.26 | 35.49 | 0.008 |
| **Line × Year** | 320 | 2031.0 | 6.35 | 2.19 | <0.001 | 5959.9 | 18.62 | 2.07 | <0.001 | 4775.4 | 14.92 | 1.89 | <0.001 | 13537 | 42.3 | 2.31 | <0.001 |

**Supplementary Table S2**. Analysis of variance for early-season, mid-season, late-season and total fruit yield (kg/plot; 5 plants per plot) in the validation population. Genotype was treated as a fixed effect and replication as a random effect in a linear mixed model fitted by REML. SS=sum of squares; MS= Mean squares; df= degree of freedom; F= F-statistics; P=probability value. Degree of freedom for the genotype effect were estimated using Satterthwaite’s approximation (denominator df: early = 54.05, mid= 52.17, late = 53.75, total = 53.16).

| **Source of variation** | **df** | **Early-season yield** | | | | **Mid-season yield** | | | | **Late-season yield** | | | | **Total yield** | | | |
| --- | --- | --- | --- | --- | --- | --- | --- | --- | --- | --- | --- | --- | --- | --- | --- | --- | --- |
|  |  | **SS** | **MS** | **F** | **P** | **SS** | **MS** | **F** | **P** | **SS** | **MS** | **F** | **P** | **SS** | **MS** | **F** | **P** |
| **Genotype** | 9 | 553.1 | 61.45 | 13.16 | <0.001 | 39.18 | 4.35 | 1.97 | 0.06 | 59.06 | 6.56 | 1.29 | 0.26 | 842.16 | 93.57 | 14.78 | <0.001 |

**Supplementary Table S3**. Estimated marginal means, standard errors (SE), and Tukey mean separation groups for early-season, mid-season, late-season and total fruit yield (kg/plot; 5 plants per plot) across genotype classes in the validation population. Means and SE were obtained from linear mixed models with genotype as a fixed effect and replication as a random effect. Means sharing the same letter within a column are not significantly different at α= 0.05 (Tukey’s HSD).

| Genotype | Early season fruit yield (kg/plot) | | | Mid-season fruit yield (kg/plot) | | | Late-season fruit yield (kg/plot) | | | Total fruit yield (kg/plot) | | |
| --- | --- | --- | --- | --- | --- | --- | --- | --- | --- | --- | --- | --- |
|  | Mean | SE | Group | Mean | SE | Group | Mean | SE | Group | Mean | SE | Group |
| BC1F2 Tolerant | 8.50 | 0.869 | cde | 5.34 | 0.684 | a | 0.45 | 0.917 | a | 16.26 | 1.14 | cd |
| BC1F2 Heterozygous | 3.94 | 0.813 | ab | 5.22 | 0.650 | a | 4.85 | 0.860 | a | 14.01 | 1.08 | bc |
| BC1F2 Susceptible | 1.82 | 1.14 | a | 0.73 | 0.967 | a | 4.79 | 1.2 | a | 8.33 | 1.43 | a |
| BC2F2 Tolerant | 6.46 | 0.868 | bcde | 5.13 | 0.684 | a | 3.21 | 0.916 | a | 14.86 | 1.14 | bc |
| BC2F2 Heterozygous | 5.18 | 0.813 | abc | 4.55 | 0.650 | a | 3.08 | 0.86 | a | 12.81 | 1.08 | abc |
| BC2F2 Susceptible | 4.67 | 0.813 | ab | 4.94 | 0.650 | a | 3.35 | 0.86 | a | 12.96 | 1.08 | abc |
| Fla. 8044 | 9.47 | 0.813 | ef | 5.79 | 0.650 | a | 4.25 | 0.86 | a | 19.52 | 1.08 | d |
| Fla. 8059 | 5.28 | 0.868 | abcd | 3.48 | 0.684 | a | 2.41 | 0.916 | a | 11.22 | 1.14 | ab |
| HTRIL-076 | 12.77 | 0.936 | f | 4.99 | 0.726 | a | 3.15 | 0.986 | a | 20.81 | 1.21 | d |
| BC1F1 | 8.98 | 0.869 | def | 5.45 | 0.684 | a | 5 | 0.917 | a | 19.40 | 1.14 | d |
